# Editorial

**Published:** 1914-04

**Authors:** 


### EDITORIAL

The Business Office of the *Journal-Record* is 23 West Alabama Street.

The Editorial Office is 1014-15 Atlanta National Bank Building.

Address all Business Communications to *Journal-Record of Medicine*, 23 West Alabama Street.

Make remittances payable to *The Journal-Record of Medicine.*

On matters pertaining to the Editorial and Original Communications, address Edgar G. Ballenger, M. D., Atlanta, Ga.

Reprints of Original Articles will be furnished at cost price. Requests for the same should always be made in THE MANUSCRIPT.

We will present, postpaid, on request to each contributor of an original article, twenty (20) marked copies of *The Journal-Record of Medicine* containing such article.

### THE ATLANTA MEETING OF THE MEDICAL ASSOCIATION OF GEORGIA.

The sixty-fifth annual session of the Medical Association of Georgia, was held in Atlanta April 15, 16 and 17. The attendance was the largest on record, there being more than 400 registered. The program was finished nearly on time at every session. There were many interesting papers read and followed by lively discussions. Dr. Ralston Lattimore made a splendid president.

The association adopted several important resolutions. Two were indorsements of bills which are shortly to come before the legislature—the public health bill and the vital statistics bill. The public health bill provides for a board of health for each county, under the supervision of the secretary of the state board of health; sanitary and health officers in each county; health inspection of school children, and sanitary inspection of public buildings. The vital statictics bill provides for systematic complication of the birth, death, marriage, and general living statistics of the state. It was stated that Georgia was one of the five states in the Union which does not possess such a law.

Resolutions of thanks were also offered the Fulton County Medical society for the banquet tendered the association at the Hotel Ansley Thursday night.

The election of officers for the forth-coming year resulted as follows: Dr. W. B. Hardman, Commerce, Ga., President; Dr. H. J. Williams, Macon, Vice-President; Dr. Patterson, Second Vice-President and Dr. W. C. Lyle, Augusta, Secretary and Treasurer. Dr. E. C. Davis, of Atlanta, was elected delegate to the convention of the American Medical Association, which meets in June at Atlantic City.

Macon was chosen as the next meeting place.

